# Evaluation of the Effect of Cariprazine on Memory and Cognition in Experimental Rodent Models

**DOI:** 10.3390/ijerph192214748

**Published:** 2022-11-09

**Authors:** Hristina Ivanova Zlatanova, Maria Todorova Georgieva-Kotetarova, Natalia Borisova Vilmosh, Ilin Kostadinov Kandilarov

**Affiliations:** Department of Pharmacology and Clinical Pharmacology, Faculty of Medicine, Medical University Plovdiv, 4002 Plovdiv, Bulgaria

**Keywords:** cariprazine, scopolamine, recognition, maze learning, spatial memory, avoidance learning

## Abstract

The main symptoms of schizophrenia are categorized as positive, negative, and cognitive. Cognitive impairments do not generally respond to antipsychotics. Cariprazine is a novel antipsychotic conceived with the idea that high affinity for D3 receptors may elicit a favorable response in the management of cognitive deficits. We evaluated the pro-cognitive properties of 14-day long pre-treatment with cariprazine (0.25, 0.5, and 1 mg/kg b.w. intraperitoneally) in experimental rodent models with scopolamine-induced memory impairment employing novel object recognition test (NORT), T-maze, Y-maze, and passive avoidance tasks (step-through and step-down). Statistical analysis was performed with One Way ANOVA. In NORT cariprazine increased the recognition index. In T-maze and Y-maze cariprazine increased the working memory index as well as the percentage of spontaneous alternation. Cariprazine improved learning and memory in both short-term and long-term memory retention tests in step-down and step-through tasks. Cariprazine improves learning, recognition, and spatial memory in rats with scopolamine-induced memory impairment. Cariprazine’s beneficial effect on cognition is likely due to its affinity for D3 receptors, as well as agonism at 5-HT1A receptors. Most probably, the cognitive-enhancing properties of cariprazine are the result of integrated modulation in the amygdala, hippocampus, and prefrontal cortex.

## 1. Introduction

Schizophrenia is a chronic mental disorder mainly characterized by episodes of psychosis [1,2]. The leading hypothesis regarding the pathophysiology of schizophrenia and similar psychotic disorders is the dopaminergic theory—abnormal dopamine signaling in the prefrontal cortex, mesolimbic and mesocortical pathways [3]. The main symptoms of schizophrenia are categorized as positive (e.g., delusions, hallucinations), negative (e.g., apathy, anhedonia, asociality), and cognitive (e.g., memory impairment, affected problem solving) [4].

Conventional antipsychotics, such as chlorpromazine or haloperidol, reduce positive symptoms by blocking dopamine D2 receptors in the mesocortical pathway [5,6,7]. The D2 receptor inhibition in other brain areas, however, leads to adverse drug reactions, such as cognitive impairment, movement disorders, endocrine imbalances. Conventional antipsychotics not only do not mitigate but may even aggravate negative symptoms observed in psychotic disorders, such as blunted affect, apathy, social withdrawal, and anhedonia [8,9]. Atypical or second-generation antipsychotics are favored because they diminish both positive and negative symptoms by antagonizing D2 and serotonergic 5-HT2A receptors. They are unfortunately linked with metabolic side effects with high incidence, e.g., hyperlipidemia, weight gain, diabetes mellitus [10,11]. Cognitive impairments do not generally respond to antipsychotics [12].

Dopamine D3 receptors are assumed to contribute to the development of negative and cognitive symptoms [13,14]. Nucleus accumbens is one of the principal locations of D3 receptors. They are also found, albeit with lower density in the thalamus, hippocampus, and cortex, all areas crucial in the development of negative symptoms and the cognitive deficit connected to schizophrenia [15]. The D3 receptor is considered to play an important role in mood and cognition and has come into view as a prospective pharmacological target for neuropsychiatric disorders [16,17].

Cariprazine is a novel antipsychotic medication manufactured by Forest Laboratories (New York, NY, USA) and approved by the Food and Drug Administration in September 2015 [18]. It is a dopamine-serotonin partial agonist in the same pharmacologic class as brexpiprazole and aripiprazole [19], although it shows agonistic/antagonistic properties depending on the endogenous dopamine levels [20]. Cariprazine was conceived with the idea that high affinity for D3 receptors may elicit a favorable response in the management of affective and cognitive deficits connected with schizophrenia and bipolar disorder [21,22,23].

In recent animal studies, cariprazine has demonstrated pro-cognitive activity. It enhanced cognition significantly in scopolamine-treated rats in the water maze test [24]. In an animal model of PCP-induced cognitive impairment, pre-treatment with cariprazine markedly lessened the PCP-triggered cognitive deficits [25,26]

The aim of this study is to evaluate the pro-cognitive properties of cariprazine in experimental rodent models with scopolamine-induced memory impairment involving both short- and long-term, spatial and recognition memory tests.

## 2. Materials and Methods

All experiments were approved by the Animal Health and Welfare Directorate of the Bulgarian Food Safety Agency and are in accordance with Permit No. 255, based on the position of the Ethics Committee, Bulgarian Food Safety Agency, Protocol No. 171/08. 10. 2019.

### 2.1. Test Substances

Reagents used in the experiments were cariprazine hydrochloride (Gedeon Richter Plc., Hungary), NaCl 0.9% (Sopharma AD, Sofia, Bulgaria), scopolamine (Merck, Darmstadt, Germany). The cariprazine was dissolved in saline and administered intraperitoneally for 14 days before testing.

### 2.2. Animals

All experiments were performed on 6-week-old male Wistar rats (n = 50) weighing approx. 200 g, randomly divided into 5 parallel experimental groups, each consisting of 10 animals. Groups 1 and 2 were administered saline solution, the other three groups received cariprazine in increasing doses of 0.25, 0.5, and 1 mg/kg b.w. The rats were housed in a temperature-controlled environment on a light/dark cycle of 12/12 h with free access to food and water.

### 2.3. Experimental Models

#### 2.3.1. Memory Impairment Model

Memory impairment was induced on the days of the experiments 30 min after treatment with saline and cariprazine by applying scopolamine (SCP) 1 mg/kg b.w. intraperitoneally (excepting group 1—negative control) [27].

#### 2.3.2. Novel Object Recognition Task (NORT)

The NORT was conducted in an open-field arena. On the first day, the animals were placed in the field with two identical objects positioned at an equal distance. On the consecutive day, the rats were allowed to explore the open field in the presence of a familiar object, and a novel object to test recognition memory. The time spent exploring each object and the recognition index (*RI*) percentage were recorded [28].

$$RI=\frac{N}{N+F}\times 100$$

*N*—time spent studying the novel object

*F*—time spent studying the familiar object

#### 2.3.3. T-Maze (Spatial Working Memory) Test

We used a maze in the shape of the capital letter “T”, positioned 50 cm above ground, with a stem length of 50 cm and an arm length of 40 cm. Animals were initially placed in the stem of the T-Maze. Upon leaving the stem, they chose between entering either the left or the right arm. The test relies on either spontaneous or rewarded alternation. In our study, we used the latter. Animals were left without food for 24 h prior to the experiment, which consisted of 11 trials—an initial forced trial, followed by 10 choice trials with 5-min intertrial time. During the forced trial, one of the arms was barred, and reward pallets were placed in the opposite arm. During choice trials, both arms were accessible, and the food was located in the same arm as in the 1st trial. Working memory index was calculated (*WMI*) [29,30].

$$WMI=\frac{CC}{N}\times 100$$

*CC*—correct choices

*N*—total number of trials

#### 2.3.4. Y-Maze Spontaneous Alternation Test

We used a Y-shaped maze with three opaque arms positioned at a 120° angle from each other. The animal was placed in the center of the maze and then allowed to freely explore the three arms for a duration of 5 min. The number of arm entries and the number of triads were recorded to calculate the percentage of spontaneous alternation (*SA*) [29,30].

$$SA=\frac{T}{N-2}\times 100$$

*T*—number of triads

*N*—total number of entries

#### 2.3.5. Step-Down Passive Avoidance Test

We used an automatic device (Ugo Basile, Italy) comprised of a clear acrylic chamber with an electrified grid floor and an elevated platform in the center. The training session consisted of two consecutive days. The rats were placed on the platform, and if they stepped down from it, they received an electric shock (10 sec duration, intensity 0.4 mA). Short-term memory was assessed 24 h later, and a long-term memory test was performed on the 8th day. Animals remaining on the platform for longer than 60 s (latency time) were considered trained. The memory retention tests were conducted without the electrical shock. Both learning and retention tests consisted of two trials [31,32].

#### 2.3.6. Step-Through Passive Avoidance Test

We used an automatic device (Ugo Basile, Italy), consisting of a light and a dark chamber connected by a sliding door. The training session was performed on two consecutive days. The rats were placed in the light chamber, and following a 7-s door delay, they gained access to the dark compartment. When entering the dark compartment (training latency), the door closed, and the rat was subjected to a brief aversive stimulus (electrical foot shock, 9-sec duration, intensity 0.4 mA). Short-term memory was appraised on the 3rd day and long-term memory on the 10th day, with animals that remained in the lit chamber for longer than 178 s being considered as trained. The memory retention tests were performed without the electrical shock. Both learning and retention tests consisted of three trials [33,34].

### 2.4. Statistical Analysis

Statistical evaluation was executed with IBM SPSS 20.0 software employing One Way ANOVA, Tukey, and Games–Howell post hoc depending on Levene’s test for equal variances. The normality of distribution was determined with Shapiro–Wilk test. Results are given as arithmetic mean and standard error of the mean (mean ± SEM). *p*-value ≤ 0.05 was deemed to be statistically significant.

## 3. Results

### 3.1. NORT

**Figure 1:**
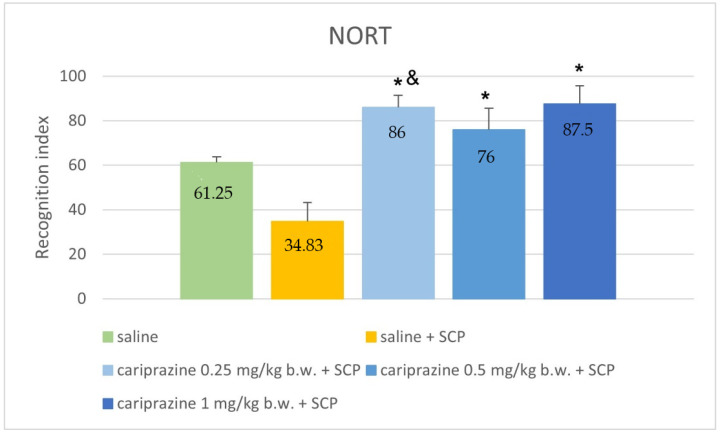
Comparison of recognition index values (percentage) between control groups and groups treated with cariprazine in NORT. SCP—scopolamine * *p* ≤ 0.05 compared to saline + SCP, & *p* ≤ 0.05 compared to saline.

In the Novel object recognition test, we observed significant differences between the groups (*p* < 0.001, ANOVA). All three groups, treated with cariprazine (in doses of 0.25, 0.5, and 1 mg/kg b.w.) markedly increased the recognition index compared to the control group treated with saline solution and scopolamine (*p* = 0.004, *p* = 0.046, and *p* = 0.006, respectively; Games–Howell post hoc). Cariprazine at a dose of 0.25 mg/kg b.w. also showed a significant difference with the rats treated only with saline without added scopolamine. The results are graphically represented in Figure 1.

### 3.2. T-Maze and Y-Maze

**Figure 2:**
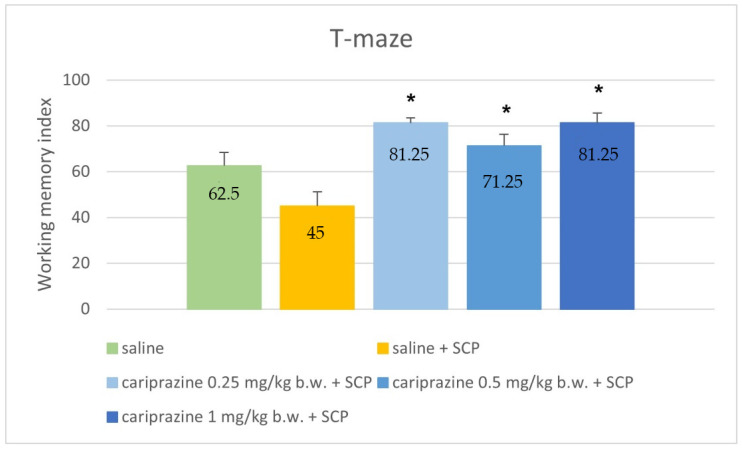
Comparison of working memory index values (percentage) between control groups and groups treated with cariprazine in the T-maze task. SCP—scopolamine * *p* ≤ 0.05 compared to saline + SCP.

The working memory index values in the T-maze task varied significantly between the experimental groups (*p* < 0.001, ANOVA). All rats treated with cariprazine (in doses of 0.25, 0.5, and 1 mg/kg b.w.) showed a notable increase in the WMI, compared to the control group treated with saline solution and scopolamine (*p* < 0.001, *p* = 0.008, and *p* < 0.001, respectively; Tukey post hoc). The results are graphically represented in Figure 2.

**Figure 3:**
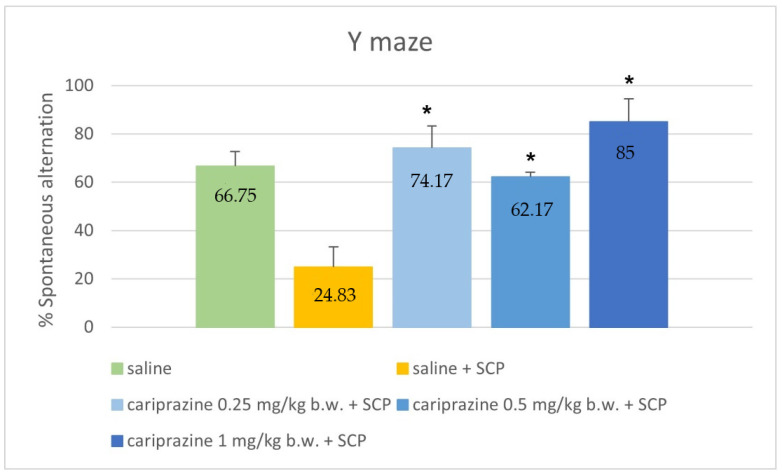
Comparison of spontaneous alternation values (percentage) between control groups and groups treated with cariprazine in the Y-maze task. SCP—scopolamine * *p* ≤ 0.05 compared to saline + SCP.

The Y-maze test showed similarly significant differences between the groups (*p* < 0.001, ANOVA). The results of this task demonstrated that there is a marked increase in the percentage SA in the groups, treated with capriprazine (*p* = 0.017, *p* = 0.030 and *p* = 0.006, respectively; Games–Howell post hoc), compared to the saline + scopolamine group. The results are graphically represented in Figure 3.

### 3.3. Step-Down and Step-Through Tests

The experimental groups in both passive avoidance tasks (step-down and step-through) demonstrated statistically significant differences (*p* < 0.001, ANOVA, in both short-term and long-term memory retention tests). Pre-treatment with cariprazine in all doses used in our experiment significantly increased latency values compared to the groups treated with saline and scopolamine. These findings were observed on the 3rd day of testing (short-term memory evaluation), as well as on the 8th (for step-down) and 10th (for step-through) day of testing (long-term memory evaluation). The results are represented in detail in Table 1 and Table 2.

## 4. Discussion

Cariprazine is a novel antipsychotic medication that has been on the pharmaceutical market for several years. Cognitive symptoms are typical in schizophrenic patients and are resistant to antipsychotic medication [35,36]. Our study aimed at assessing cariprazine’s effect on cognition and memory in experimental rodent models.

Cariprazine is unique in its mechanism of action. Although it belongs to the same class as aripiprazole and brexpiprazole, it shows significant differences in its affinity for dopamine receptors. Cariprazine acts as a partial agonist at dopamine D2 and D3 receptors and serotonin 5-HT1A receptors and antagonist at 5-HT2A receptors [37]. In vitro studies demonstrate that cariprazine has nearly ten times higher affinity for the human dopamine D3 than D2 receptor [24,38]. Cariprazine binds highly to D2 and D3 receptors in a stable manner, both in rodents and schizophrenic patients [20,35]. Cariprazine is singular in its ability to bind D3 receptors with higher affinity than even dopamine, which in essence, leads to a blockade of D3 receptors [39].

Dopamine is instrumental in modulating synaptic plasticity and information storage [37]. A lot of recent data point towards D3 receptors as a major factor in the processes of cognition and memory. Nakajima et al. suggest that D3 receptors in vivo are occupied by dopamine for extensive periods of time. The authors propose, therefore, that D3 receptors are crucial modulators of normal dopaminergic function and, consequently, cognition. D3 receptors are located in the ventral striatum (nucleus accumbens) and other limbic areas. D3 receptor levels are low in the dorsal striatum and several cortical regions, including the frontal cortex in humans. The dorsal striatum, however, almost completely lacks D3 receptors in rodents. D3 receptors probably regulate cortical control of cognitive functions through their inhibitory effect on mesocortical dopaminergic activity [40]. D3 receptor knockout mice demonstrated greater learning performance [24]. The particular brain areas where these receptors are expressed as well as the studies in mice with genetically deleted D3 receptors further support the hypothesized role of D3 receptors in cognition [41]. D3 receptor-selective antagonists have shown cognitive-enhancing capability [24]. Watson et al., also propound that D3 receptor blockade can improve cognition [35].

Based on cariprazine’s high affinity and preference for D3 versus D2 receptors, it is therefore expected to demonstrate pro-cognitive activity. Gyertyán et al. found that cariprazine enhanced cognition in a scopolamine-induced learning impairment model. They observed similar results with two D3 receptor-preferring D3/D2 antagonists, S33138 and RG-15 [24]. In another study in rodents, cariprazine up-regulated D3 receptors, which the authors suggested could also provide alleviation of depression and negative symptoms [11]. Stahl et al., hypothesized that cariprazine’s partial agonist activity at presynaptic D3 receptors in the ventral tegmental area disinhibits dopamine release in the prefrontal cortex, thus establishing positive dopamine tone which improved mood and had anti-anhedonia effects in animal models [42].

In our study, cariprazine demonstrated statistically significant improvement in learning and memory in rats in all scopolamine-induced memory impairment models that we employed.

The novel object recognition test is used to assess cognition, particularly recognition memory, in rodent models of CNS disorders. The NOR task shows good prognostic capability for novel antipsychotic agents [26,30,43]. The principal advantage of NORT is that it depends on rodents’ innate propensity for investigating novelty. It does not require numerous training sessions or any type of reinforcement to provoke behavior [44]. While it is simple and quick, since there is only one training session, it is not possible to assess potential variability in the rate of learning [28]. In the rat brain, the perirhinal cortex is essential for recognizing a previously encountered object as familiar. The medial temporal lobe plays a vital role in recognition memory formation. Studies with rodents have demonstrated that for visual object recognition memory, the parahippocampal regions of the temporal lobe are of major importance [44,45,46,47]. In all doses used in the present study, cariprazine increased the recognition index. This parameter was even higher than the one recorded for the control group with saline and no added scopolamine, although no statistical significance was reached. Our findings are in accord with those of Watson et al., who demonstrated that in adult male rats, cariprazine caused a dose-dependent reversal of a delay-induced impairment in NORT [35]. Neill et al., however, did not observe significant improvement in PCP-induced impairment in NORT. The highest dose of cariprazine the authors used was 0.25 mg/kg, which could account for the difference with our results [26].

T-Maze and Y-Maze tasks are behavioral tests for evaluating investigative behavior in rodents, more specifically, spatial memory. These tests are based on the readiness of rodents to explore a new territory, and they usually prefer to examine a new arm of the maze rather than going back to one that was already visited. The hippocampus, septum, basal forebrain, and prefrontal cortex are involved in these tasks [30,48]. Hafting et al. discovered that the entorhinal cortex contains a neural map of the spatial environment in rats [49]. The T-maze task is one of the simplest tools to assess spatial working memory and yields highly reproducible results. The major drawback, however, is that it has one choice point with just two alternatives, which greatly increases the chance of success. The rats may also solve the maze by applying a strategy other than spatial. The T-maze necessitates continual handling of animals, which may cause stress and influence the results. The Y-maze is similar to the T-maze. The only difference is that the arms in the Y-maze call for a gradual turn, unlike the sharp turns in the T-maze, which might decrease learning time in the Y-maze [29]. Cariprazine increased both the working memory index as well as the percentage of spontaneous alternation. Similarly to NORT, the observed values for the groups treated with cariprazine were higher than the control rats treated only with saline. Zimnisky et al. reported similar results. In their model of PCP-induced memory impairment, cariprazine mitigated the effects of PCP on spatial working memory [25].

The passive avoidance tests (both step-down and step-through tasks) are fear-exacerbated tests used to gauge learning, short-term and long-term memory. In these tests, rats learn to avoid an environment in which an unpleasant stimulus (e.g., a foot-shock) was formerly administered. Rodents with normal learning and memory will keep away from entering the dark compartment or avoid stepping down from the platform, in which cases, they had previously been exposed to the shock [30,43]. These tasks, however, have not proved to be dependable and contradictory results with the same drugs have been observed. The tests have also been criticized for not considering non-memory factors such as shock sensitivity, emotionality, and unprompted locomotion [50]. Another issue is that it is difficult to correlate passive avoidance tasks and human memory tasks [51]. Several studies have shown that the amygdala has a crucial role in the acquisition and retention of this type of learning. Passive avoidance tests rely on the coordinated activity of CA1 pyramidal cells, the entorhinal cortex, and the posterior parietal cortex regulated by the amygdala [52]. Along with the amygdala, the hippocampus plays a major part in the acquisition and retention of passive avoidance learning [53]. This is probably due to the critical role contextual conditioning plays in passive avoidance models [54]. Cariprazine improved learning and memory retention rates in both short-term and long-term memory retention tests. We could not find any published articles that employed passive avoidance tasks to evaluate cariprazine’s effect on learning with which to compare our results.

In our study, cariprazine improved learning associated with various brain regions, including the hippocampus, amygdala, prefrontal cortex. Similar findings were observed by Barnes et al. whose experiments cariprazine demonstrated broad effects on numerous cognitive domains [36]. Caccia et al., observed that long-term intraperitoneal administration of cariprazine caused adaptive changes in dopaminergic, serotonergic, and glutamatergic receptors in a variety of rat brain regions. An increase in D2 receptors was noted in the medial prefrontal cortex, nucleus accumbens, and hippocampus [55,56]. In the rat substantia nigra, cariprazine partially inhibited dopamine activity after cumulative dosing, an effect thought to be due to partial agonism of midbrain autosomal D2 receptors [57]. Huang et al. described similar results—cariprazine increased dopamine, norepinephrine, serotonin, glutamate, and glycine efflux in the rat nucleus accumbens and hippocampus [15]. In the experiment described by Caccia et al., cariprazine increased 5-HT1A receptor density in the rat hippocampus and in the medial and dorsal prefrontal cortex. Agonism or partial agonism at 5-HT1A receptors may play a part in cariprazine’s ability to improve cognitive deficits in animal models [55]. Citrome et al. observed comparable findings regarding the agonism at serotonin 5-HT1A receptors [58]. Herman et al. also registered that in the dorsal raphe nucleus cariprazine acted as an agonist at the 5-HT1A autoreceptors. Agonism at 5-HT1A receptors has been shown to contribute to the regulation of mood and cognition [59]. However, Kehr et al. observed that Cariprazine 0.2 mg/kg had no effect on dopamine, norepinephrine, or serotonin levels, which could be explained by the dose used by the authors. In the same study, a higher dose of 0.8 mg/kg ameliorated PCP-induced cognitive deficits [60].

In the present study, cariprazine was administered systemically, and therefore it is not possible to identify the exact brain region that underlies cariprazine’s positive effects on cognitive impairment. The tests employed in our experiment point towards the amygdala, hippocampus, and prefrontal cortex. Most probably, the cognitive-enhancing properties of this antipsychotic are the result of integrated modulation in all these areas. Cariprazine’s beneficial effect on cognition is likely due to its affinity for D3 receptors, as well as agonism at 5-HT1A receptors in the amygdala, hippocampus, and prefrontal cortex.

## 5. Conclusions

Pre-treatment with cariprazine improves learning, recognition, and spatial memory in rats with scopolamine-induced memory impairment. The research findings were consistent in all the tests employed in this study—NORT, T-maze, Y-maze, passive avoidance tasks (step-down and step-through). The results did not vary significantly between the doses of cariprazine that we used—0.25, 0.5, and 1 mg/kg b.w.

## Figures and Tables

**Table 1:** Training latency values (in seconds) in step-down passive avoidance test.

Group	Mean ± SEM	*p*	Mean_1_ ± SEM_1_	*p*_1_
Saline	52.90 ± 3.65	<0.001 *	53.76 ± 3.58	<0.001 *
Saline + SCP	7.29 ± 0.62	-	8.49 ± 1.03	-
Cariprazine 0.25 mg/kg b.w. + SCP	32.73 ± 3.36	0.001 *	28.16 ± 4.00	0.031 *
Cariprazine 0.5 mg/kg b.w. + SCP	36.55 ± 2.99	<0.001 *	33.68 ± 5.36	0.013 *
Cariprazine 1 mg/kg b.w. + SCP	34.51 ± 3.76	0.001 *	38.63 ± 6.38	0.012 *

* Games–Howell post hoc; mean ± SEM, *p*-values on 3rd day retention test; mean_1_ ± SEM_1_, *p*_1_-values on 8th day retention test; * *p* ≤ 0.05 compared to saline + SCP. SCP—scopolamine; SEM—standard error of the mean.

**Table 2:** Training latency values (in seconds) in step-through passive avoidance test.

Group	Mean ± SEM	*p*	Mean_1_ ± SEM_1_	*p*_1_
Saline	175.20 ± 2.62	<0.001 *	177.73 ± 0.28	<0.001 *
Saline + SCP	30.28 ± 5.20	-	54.50 ± 7.20	-
Cariprazine 0.25 mg/kg b.w. + SCP	137.30 ± 14.97	0.001 *	153.60 ± 16.22	0.002 *
Cariprazine 0.5 mg/kg b.w. + SCP	140.13 ± 12.44	<0.001 *	149.60 ± 18.59	0.007 *
Cariprazine 1 mg/kg b.w. + SCP	146.39 ± 15.44	<0.001 *	164.01 ± 13.99	<0.001 *

* Games–Howell post hoc; mean ± SEM, *p*-values on 3rd day retention test; mean_1_ ± SEM_1_, *p*_1_-values on 10th day retention test; * *p* ≤ 0.05 compared to saline + SCP. SCP—scopolamine; SEM—standard error of the mean.

## Data Availability

All data generated or analyzed during this study are included in this published article.
